# Influence of Adipose Tissue-Derived Stem Cells on the Burn Wound Healing Process

**DOI:** 10.1155/2019/2340725

**Published:** 2019-02-11

**Authors:** Claudio Luciano Franck, Alexandra Cristina Senegaglia, Lidiane Maria Boldrini Leite, Sérgio Adriane Bezerra de Moura, Nathália Forti Francisco, Jurandir Marcondes Ribas Filho

**Affiliations:** ^1^Evangelical Faculty of Paraná, Brazil; ^2^Core for Cell Technology-Pontifical Catholic University of Paraná, Brazil; ^3^Department of Morphology-Federal University of Rio Grande do Norte, Brazil

## Abstract

Burns are lesions in which the thermal energy of the causative agent transfers heat to the surface of the body, causing superficial or deep damage to the skin with protein denaturation in cells and biochemical maladjustments, which delay and disrupt the cicatricial process, increasing the chances of functional and aesthetic sequelae. This study evaluates the influence of adipose tissue-derived stem cells (ADSCs) on burn healing in terms of the size of the cicatricial space and quantified measures of collagen deposition, inflammatory infiltrate, blood vessels, and lymphatic vessels. Initially, intra-abdominal adipose tissue was resected from a single donor Wistar rat that was not part of any of the subsequent groups to obtain ADSCs by isolation and cell culture. Burns were made in the left lateral abdominal region of Wistar rats by contact with a square ceramic paper with a 484 mm^2^ area heated to 100°C for 30 seconds. Intradermal ADSC transplantation was performed in two stages. The first was on the same day of the burn, when 3.2 × 10^6^ ADSCs were transplanted shortly after the burned region cooled, while the second stage occurred four days later with the same number of ADSCs. The progress was evaluated by immunohistochemical methods and H&E, Masson's trichrome, Picrosirius red, and Lyve-1 immunofluorescence staining. Despite the quantitative similarity of blood vessels and the inflammatory infiltrate observed by H&E, there were statistically significant differences between the groups on the fourteenth day of evolution. The group that received ADSCs showed a reduction in the scar tissue area, increased collagen type III deposition, and a quantifiable reduction in lymphatic vessels, so we conclude that ADSCs influence the healing of total thickness burns in rats.

## 1. Introduction

Full thickness burns are characterized by being a dry, inelastic lesion with a color ranging from waxy white to black, and the resolution of these burns is rare without surgical intervention [1, 2]. Several strategies are used to recover the complex skin structure, characterized by cellular diversity and three-dimensionality [3]. Tissue engineering is aimed at optimizing the aesthetic and functional reconfiguration of the skin using mesenchymal stem cells (MSCs) [4, 5]. Stem cell transplantation on burns is aimed at improving scar quality by early closure of the lesion to accelerate the cicatricial process, preventing contractures and cicatricial formations, regenerating the skin and its appendages and attenuating inflammation [6]. The therapeutic interest in the use of MSCs in healing derives from the ability of these cells to differentiate into several cell lines with low immunogenicity and the production of paracrine substances [7], which distinctly benefit each of the cicatricial phases, interfering with cellular mobilization [4, 8]. Adipose tissue is an abundant and accessible source of multipotent adult stem cells [9]. After processing of adipose tissue, the stromal vascular fraction (SVF) is obtained; from this heterogeneous cell set, it is possible to isolate and cultivate ADSCs, which can differentiate into mesodermal, ectodermal, and endodermal cells [10]. The interaction of ADSCs with M2 macrophages promotes the release of VEGF and IL-10 by macrophages, along with VEGF, HGF, and FGF-b release [9], leading to angiogenic, lymphangiogenic, and anti-inflammatory effects [9, 10].

The aim of this study was to evaluate whether intradermal transplantation of ADSCs could influence the cicatricial process in an experimental model of thermal burns in rats. Evaluations were performed on the fourteenth day of evolution to compare the size of the scar area and to quantify the collagen deposition, inflammatory infiltrate, blood vessels, and lymphatic vessels.

## 2. Material and Methods

This research was approved by the Ethics Committee on the Use of Animals of the Evangelical Faculty of Paraná (number 3250/2015).

### 2.1. Isolation and Cell Expansion of ADSCs

Twenty-three ninety days old Wistar male rats (*Rattus norvegicus albinus*) weighing 250–280 grams were used in the experiments. At the beginning, only one Wistar rat was used as the ADSC donor and underwent a laparotomy under general anesthesia for the dissection of adipose tissue deposits in the retroperitoneal, perirenal, and inguinal regions. These deposits were dissected bilaterally and separated from the adjacent tissues while leaving them intact by dissection via the minimum and cautious manipulation. These samples were transported under sterile conditions for subsequent cell isolation, expansion, and cryopreservation.

The adipose tissue collected under sterile conditions was washed with phosphate-buffered saline (PBS) containing 1% antibiotic solution (Gibco Invitrogen, Carlsbad, USA). The washing step was repeated until all blood vessels and connective tissues appeared to have been liberated (three washes). Adipose tissue samples were minced into small pieces and digested in 0.4% collagenase type I (Gibco Invitrogen, Carlsbad, USA) at 37°C with shaking for 30 min. After digestion, the cell suspension was filtered through a 100 *μ*m filter (BD Falcon™, Bedford, USA) for the removal of the solid aggregates. The sample was subsequently centrifuged at 450 g for 10 min at 20–22°C, and the supernatant was removed without disturbing the cells. The pellet was resuspended in 1 ml of lysis buffer (custom made) to lyse red blood cells, incubated for 10 min, washed with 10 ml of PBS+1% antibiotic solution, and centrifuged at 400 g for 10 min. The supernatant was removed, the cell pellet was resuspended in complete medium [DMEM-F12 (Gibco Invitrogen, Carlsbad, USA) with 20% fetal bovine serum (FBS) (Gibco Invitrogen, Carlsbad, USA) and 1% antibiotic solution], and the cells were cultivated in 25 cm^2^ flasks and maintained in an incubator supplied with a humidified atmosphere of 5% CO_2_ at 37°C. These cells were analyzed for their capability to differentiate into osteoblasts, adipocytes, and chondroblasts, and the immunophenotype was assessed by flow cytometry as described below.

### 2.2. Adipogenic Differentiation

The cells from passage three were cultured in adipogenic induction medium (hMSC Differentiation BulletKit; Lonza, Walkersville, MD, USA) at 37°C in 5% CO_2_/95% air and were maintained for three weeks, and the culture medium was replaced three times every seven days. Intracellular lipid droplets indicating adipogenic differentiation were confirmed by Oil Red O (Sigma-Aldrich, São Paulo, Brazil) staining with 0.5% Oil Red O in methanol and observation under an optical microscope (CK40, Olympus, São Paulo, Brazil).

### 2.3. Osteogenic Differentiation

The cells from passage three were cultured in osteogenic induction medium (hMSC Differentiation BulletKit; Lonza, Walkersville, MD, USA) at 37°C in 5% CO_2_/95% air and were maintained for three weeks, and the culture medium was replaced three times every seven days. Osteoblastic differentiation was confirmed by mineral deposition of the culture, which was assessed by Alizarin red S staining using an optical microscope (CK40, Olympus).

### 2.4. Chondrogenic Differentiation

The cells from passage three were cultured in chondrogenic induction medium (hMSC Differentiation BulletKit; Lonza, Walkersville, MD, USA) at 37°C in 5% CO_2_/95% air and were maintained for 3 weeks, and the culture medium was replaced three times every 7 days. Chondrogenic differentiation was confirmed by proteoglycan deposition, and the lacunae of the culture was assessed by Alcian blue staining using an optical microscope (CK40, Olympus).

### 2.5. Characterization of ADSCs

Immunophenotypic analysis was performed by staining 5 × 10^5^ expanded ADSCs. The cells were incubated with various conjugated monoclonal antibodies against the following antigens: CD90 (phycoerythrin (PE) conjugated), CD29 (PE conjugated), CD34 (PE conjugated), CD45 (peridinin chlorophyll protein (PerCP) conjugated), CD14 (fluorescein isothiocyanate (FITC) conjugated), and CD19 (FITC conjugated). All incubations were performed at room temperature for 30 min. Isotype-identical antibodies served as controls. After incubation, the cells were washed with PBS and fixed with PBS containing 1% paraformaldehyde. Quantitative analyses were performed using a FACSCalibur flow cytometer and FlowJo software (FlowJo, Ashland, OR, USA).

### 2.6. Thermal Lesions

Twenty-three animals were included in the study and divided into 12 in the control group (CG) and 11 in the experimental group (EG). All animals underwent general anesthesia with intramuscular application of Ketalar™ at a dose of 50 mg/kg and Virbaxyl 2%™ at a dose of 5 mg/kg. The trichotomy of the left lateral abdominal region was performed from the lower border of the costal arches to the anterior region of the left thigh with a stainless steel blade. A pattern with a 484 mm^2^ area was placed in the left abdominal region at a distance of 80 mm from the lower border of the last costal arch with one side parallel to the midline of the abdomen. The marking was carried out along the entire length of the sides of the model. A welding station (HK-936B®, Hikari) with analog temperature control was used to make the thermal lesions. A square ceramic pattern with a 484 mm^2^ area identical to that of the pattern used for the skin demarcations and with adjustable temperature was used. The temperature of the ceramic base was stipulated at 100°C at the surface that was directly in contact with the skin. To reach the desired temperature at the ceramic surface, the welding station was maintained at a constant temperature of 250°C. The temperature of the ceramic surface was monitored with an infrared digital thermometer with a laser sight (MT-320®, Minipa). By the time the temperature had reached 100°C, the device was positioned vertically on the demarcated skin. Only the pressure of the mass of the ceramic itself, which was equivalent to 54 g, was applied for a period of thirty seconds to establish the total skin thickness burn with an area of 484 mm^2^.

### 2.7. ADSC Injection in the Inflammatory and Proliferative Phase

To avoid injecting ADSCs into the still heated burned region, the temperature of the site was monitored until it cooled down. The MT-320® thermometer was used to determine the temperature of the skin immediately after the burn, and at that time, a temperature of approximately 54°C was detected. In every 30 seconds for the next five minutes, the skin cooling was monitored until the temperature reached 35°C, which corresponded to the preheating temperature.

At this moment, the EG animals, which remained under the effect of deep general anesthesia, received an intradermal injection of ADSCs. After the enzymatic dissociation, the ADSCs were conditioned in 1 ml syringes containing 0.8 × 10^6^ ADSCs each, and each animal received the volume from four syringes, totaling 3.2 × 10^6^ ADSCs. From the lines on each side of the square, a distance of 0.5 cm was measured perpendicularly from its inner surface. From these points, dotted lines were drawn parallel to the sides of the quadrilateral and thus formed a new larger square with sides of 320 mm^2^. In this new skin demarcation, the extension of each side of the square was divided into five separated points at a distance of 8 mm for the ADSC injection. The needle (13 × 0.38 mm) was inserted exactly at the demarcated points and directed at an intradermal plane until the end of the needle reached the target point.

During the slow return movement of the needle through the 8 mm line separating the stitches, a slight pressure was applied to the plunger of the syringe for the intradermal injection of 0.25 ml of the solution containing the ADSCs. This procedure was performed four times until 1 ml was injected on each side, so at the end of the transplant, a total of 4 ml, i.e., 3.2 × 10^6^ ADSCs, was administered in each Wistar rat.

On the fourth day after the burn, in the proliferative phase, the burned area of each animal was measured. These measurements were performed using a digital caliper with an analog ruler (150 mm Digital Plastic Caliper PPV 1506®, Vonder) by finding the largest distance in millimeters between the opposing sides; these distances were multiplied to obtain the total area. Then, in the eleven EG animals, the second ADSC transplant procedure was performed.

### 2.8. Histopathological Evaluation

The end of the experiment occurred on the fourteenth day after the surgical specimen was obtained. The burned area of each animal was measured by the previously mentioned method. During the burn healing process, a skin fragment was carefully removed from each animal using a no. 15 knife blade to cut the skin and perform the necropsy excision. The margins were demarcated using the square ceramic pattern with a 220 mm extension on each side.

The specimens were placed in flasks containing 10% buffered formalin (Biotec™, Pinhais, Brazil) for 48 hours. Afterward, the fragments were transferred to properly identified cassettes and sent to the Laboratory of Experimental Pathology. The tissue repair process was evaluated through histological sections stained with hematoxylin & eosin (HE), Masson's trichrome (EasyPath, São Paulo, Brazil), Picrosirius-hematoxylin (EasyPath, São Paulo, Brazil), and LYVE-1 polyclonal antibody (Bioss Antibodies Inc., Woburn, USA). The slides were scanned in the Axio Scan.Z1 equipment (Zeiss, Oberkochen, Germany) with a 20x objective, and the skin slices were analyzed in the region adjacent to the wound using the ZEN Lite software (Zeiss, Oberkochen, Germany).

By H&E staining, we evaluated the presence and distribution of inflammatory infiltrate, blood vessels, and lymphatic vessels in the lesion area, by morphological criteria. The total inflammatory cells were quantified in the central burn area in three areas: area one, which represents the borders between the epidermis and the first portion of the dermis, area two, which refers to the central region of the dermis, and area three, which constitutes the hypodermis. We use as a resource a ZEN Lite software selection tool, with a scale bar of 20 *μ*m, to aid in the counting of the inflammatory infiltrate. The total cell number of each animal was used as a comparison parameter between the study groups. The blood vessels were quantified in ten consecutive fields in the region of the superficial dermis, middle and lower, in the central area of the burn. Lymphatic vessels and small caliber veins have similarities. However, in order to differentiate them, we used as reference the endothelial cell shape and the distance between the nuclei [11]. We use as a resource an area selection tool in ZEN Lite software, with a scale bar of 50 *μ*m, to aid in vessel counting. The mean blood and lymphatic vessels of each animal were used as a comparison parameter between the study groups.

Using Masson's trichrome technique, it was possible to verify the collagen production and distribution in the region of the burned skin. Histological sections were also scanned in the Axio Scan.Z1 equipment with a 20x objective and analyzed in the region adjacent to the wound using the ZEN Lite software, and collagen was quantified with the Image Pro Plus 5.1 program (Media Cybernetics, Rockville, USA).

The staining with Picrosirius-hematoxylin determined the presence of type I and III collagen in the burned tissue matrix. For this purpose, the skin sections were evaluated in an optical microscope under polarized light (Axio Scope.A1, Zeiss, Oberkochen, Germany). Type I collagen fibers showed a red-orange coloration, while type III collagen showed a greenish coloration. Collagen fibers were quantified in the Image Pro Plus 5.1 program (Media Cybernetics, Rockville, USA) to determine the proportion of collagen types I and III in the burn region.

Lymphatic vessels quantified by H&E were subsequently qualitative identified by immunofluorescence with LYVE-1 polyclonal antibody (Bioss Antibodies Inc., Woburn, USA) conjugated to the ALEXA fluorescent molecule FLUOR™ 350.

### 2.9. Statistical Analysis

To verify the normality of the distribution of quantitative variables, D'Agostino's test was applied. For the parametric data with a normal distribution, such as the burn area, the inflammatory infiltrate, the blood and lymphatic vessels, and the evaluated collagen, Student's *t*-test was used to compare the means of the CG vs. EG. Values are expressed as the mean ± standard deviation. Statistical analyses were performed in GraphPad Prism version 5.03 for Windows (GraphPad, La Jolla, USA). The results were considered significant at *p* < 0.05.

## 3. Results

### 3.1. Isolation, Expansion, and Cell Characterization of ADSCs

**Figure 1:**
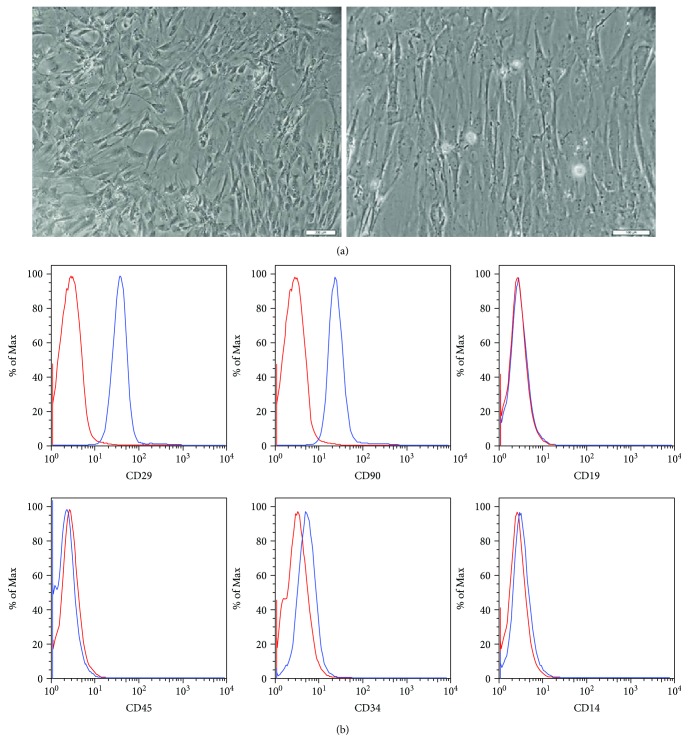
ADSCs in culture and immunophenotypic characterization. (a) Representative fields showing the fibroblast-like morphology of the ADSCs at passage 3 (magnification 40x, scale bars 200 *μ*m; magnification 200x, scale bars 100 *μ*m). (b) Representative flow cytometry analysis of cell surface markers of ADSCs at passage 3. The isotype control is shown as a red line histogram.

**Figure 2:**
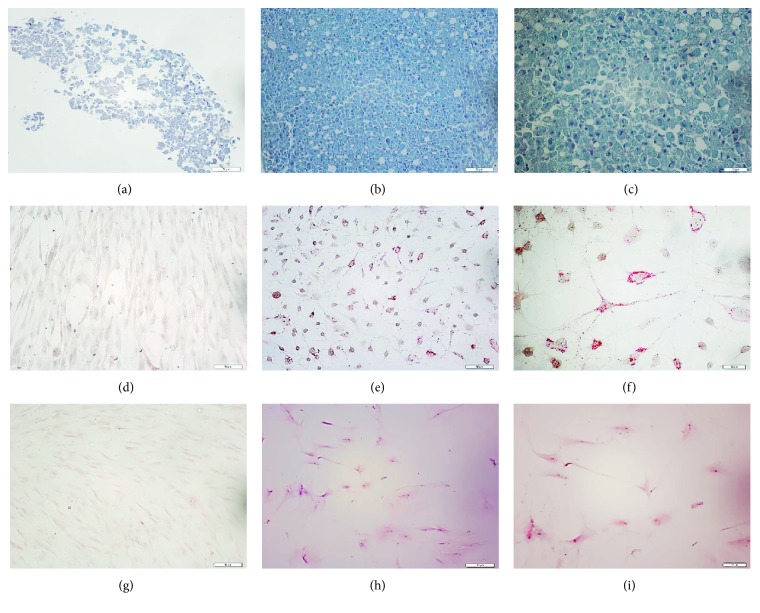
Chondrogenic differentiation (b, c) evaluated by staining with Alcian blue shows the deposition of proteoglycans and lacunae. Adipogenic differentiation (e, f) evaluated by staining with Oil Red shows the presence of lipid-rich vacuoles. Osteogenic differentiation (h, i) evaluated by staining with Alizarin red shows mineralization of the extracellular matrix. Untreated control cultures (a, d, g) without chondrogenic, adipogenic, or osteogenic differentiation stimuli are shown above each photograph. (a, b, d, e, g, h) Magnification 200x; scale bars 50 *μ*m. (c, f, i) Magnification 400x; scale bars 20 *μ*m.

After five days of cultivation, the cells that adhered to the plastic dish began growing and exhibited a fibroblast-like morphology in the subsequent passages (Figure 1(a)). The surface markers of rat adipose tissue-derived MSCs were evaluated by flow cytometry analysis. The cells were positive for the expression of ADSC-positive markers, such as CD90 and CD29 (99.2% and 99.7%), whereas the expression of ADSC-negative markers, such as CD14, CD45, CD19, and CD34, was not observed, or the number of cells with these markers was extremely low (0.39%, 0.46%, 0.28%, and 1.49%, respectively) (Figure 1(b)). The cells were positive for Alizarin red S staining, Oil Red O staining, or Alcian blue staining when the cells were cultured in osteogenic, adipogenic, or chondrogenic induction media, respectively (Figure 2). Taken together, these results indicate that these cells have phenotypic and functional characteristics of MSCs.

### 3.2. Burn Extension

**Figure 3:**
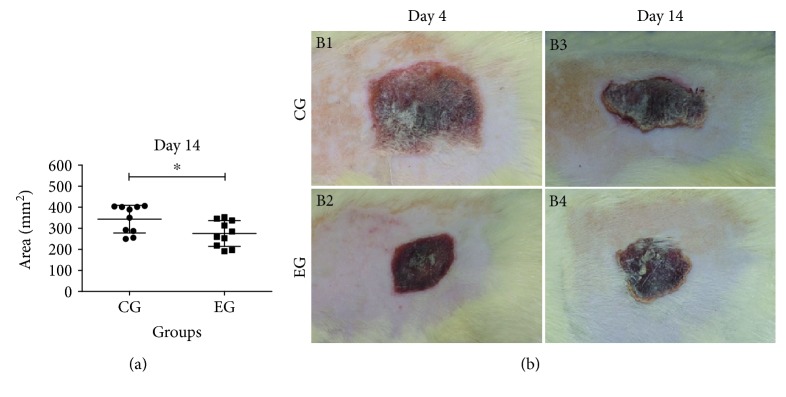
Burn healing area in rats on day fourteen. (a) Graph comparing burn areas after 14 days. (b) Images of burned areas 4 and 14 days after thermal injury. Compared to the control group (CG), the experimental group (EG) presented a significant reduction in the burn area on the fourteenth day after the first ADSC transplantation (*p* = 0.027). The results were expressed as the mean ± SD. Representative images of the burn area on the fourth (B1: CG and B2: EG) and fourteenth days after the skin was burned (B3: CG and B4: EG).

The area of scar tissue was obtained on days four, seven, and fourteen by measuring, in centimeters, the greatest distance between opposing sides, which were multiplied to calculate the area. On the 14th day after injury, EG animals that received ADSCs had a significantly smaller burn area than CG animals (275.3 ± 61.01 vs. 343.7 ± 65.70 mm^2^, *p* = 0.027) (Figure 3).

### 3.3. Inflammatory Infiltrate

**Figure 4:**
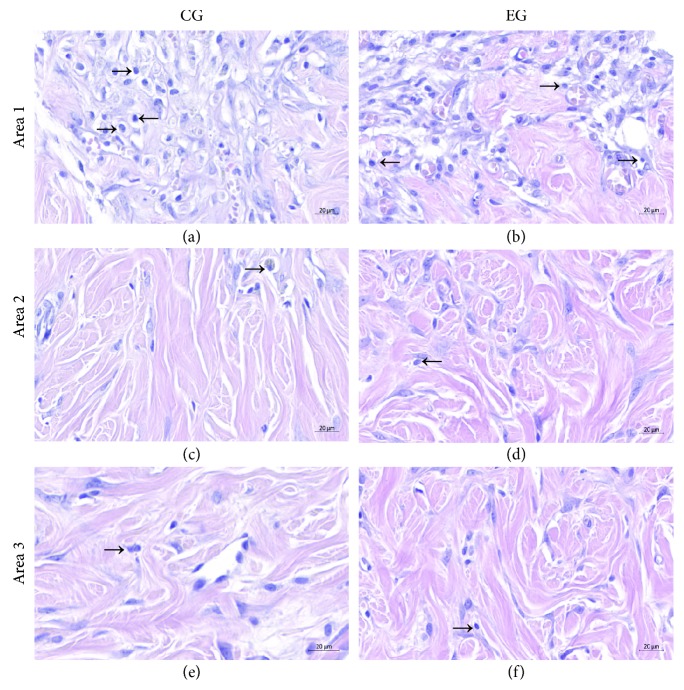
Inflammatory infiltrate present in the burn region. Inflammatory cells were quantified in the control group (CG: (a, c, e)) and experimental group (EG: (b, d, f)) in three areas: area 1 (border between the epidermis and the first portion of the dermis: (a, b)), area 2 (central region of the dermis: (c, d)), and area 3 (hypodermis: (e, f)). There was no significant difference in the inflammatory infiltrate after the ADSC treatment in the EG compared to the CG in the three areas: area 1 (*p* = 0.868), area 2 (*p* = 0.768), and area 3 (*p* = 0.378).

The presence of the total inflammatory cell count identified in each area photographed at the injury site was not significantly different between EG and CG animals in area 1, which represents the border between the epidermis and the first portion of the dermis (127.3 ± 92.34 vs. 133.3 ± 81.04, *p* = 0.868), in area 2, which refers to the central region of the dermis (33.55 ± 15.08 vs. 35.67 ± 18.56, *p* = 0.768), and in area 3, which constitutes the hypodermis (19.55 ± 8.02 vs. 22.67 ± 8.56, *p* = 0.378) (Figure 4).

### 3.4. Blood Vessels

The quantification of the blood vessels distributed in the superficial, middle, and lower dermis regions identified in each area photographed showed no significant difference between EG and CG (4.96 ± 2.11 vs. 4.43 ± 2.21, *p* = 0.563), as indicated by comparing the H&E staining to evaluate the amount of blood vessels in the scar tissue on the fourteenth day of burn evolution.

### 3.5. Lymphatic Vessels

**Figure 5:**
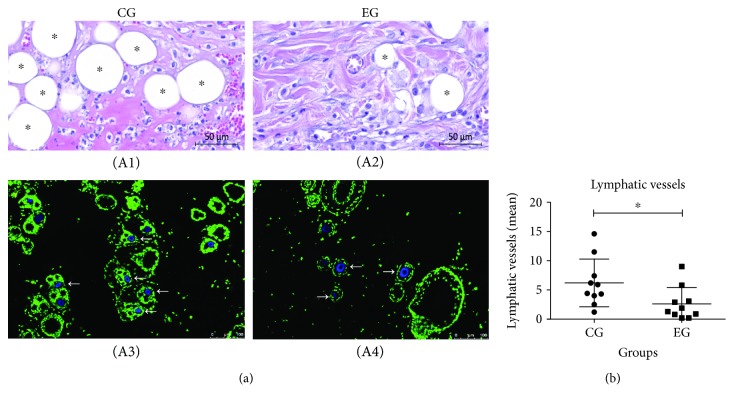
Distribution of lymphatic vessels in the burn region. (a) Quantification of lymphatic vessels by H&E (A1 and A2) and immunofluorescence staining (A3 and A4). (b) Identification of lymphatic vessels with LYVE-1 antibody by immunofluorescence staining at the lesion area (A3 and A4). (b) Comparative graph of quantified lymphatic vessels in the control group (CG) experimental group (EG). CG, which received no treatment, and EG, which was treated with ADSCs. The nuclei were stained in green and in blue the positive marking with the ligand-specific transporter trafficking between intracellular organelles (TGN) and the plasma membrane, rich in lymphatic vessels. There was a significant decrease in lymphatic vessels after treatment in the EG compared to the CG, identified by white narrows. Magnification 100x; scale bars 100 *μ*m. The data demonstrated a significant decrease in the EG compared to the CG (*p* = 0.0346). The results were expressed as the mean ± SD *μ*m.

The amount of lymphatic vessels in the scar tissue on the fourteenth day of burn evolution was evaluated with H&E. The presence of the lymphatic vessels distributed in the superficial, middle, and lower dermis regions was significantly lower in the EG animals, which received ADSC treatment, than in the CG animals, which were not treated (2.61 ± 2.81 vs. 6.20 ± 4.09, *p* = 0.0346) (Figure 5).

### 3.6. Collagen Deposition

**Figure 6:**
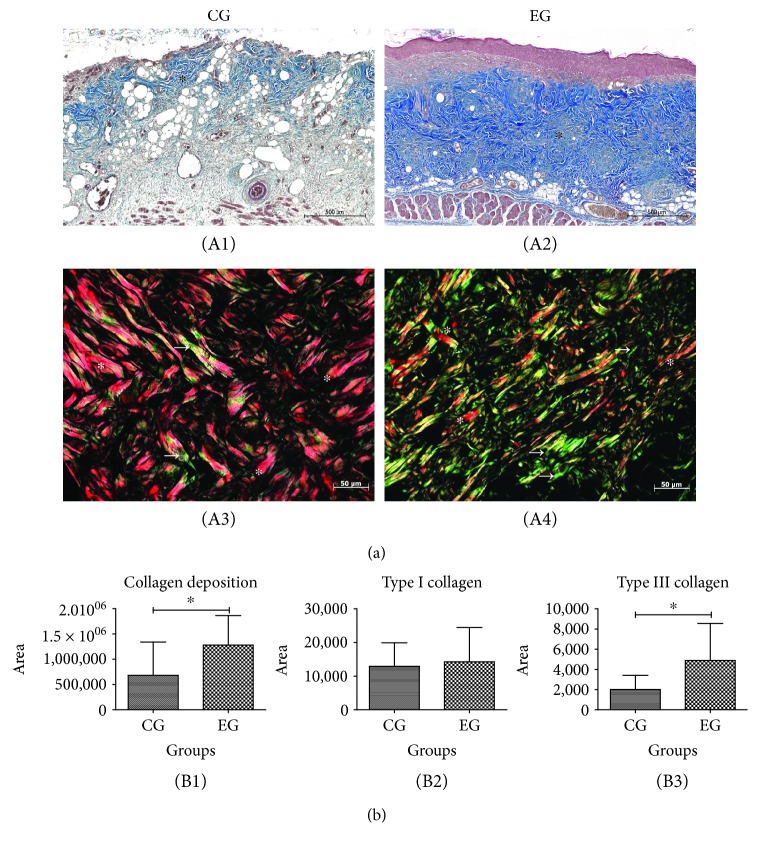
Collagen deposition in the burn region of animals subjected to thermal injury. (a) Representative images on skin cuts stained with Masson trichrome (A1 and A2) and Picrosirius-hematoxylin (A3 and A4). (b) Comparative graph of collagen deposition and type I and type III collagen in the control group (CG) and experimental group (EG). The EG (A2) presented a higher intensity of collagen fibers (^∗^) than the CG (A1). Magnification 200x; zoom: 10%; scale bars 500 *μ*m. (b) Type I (^∗^) and III (white arrows) collagen in the burn areas of CG (A3) and EG (A4) stained by Picrosirius-hematoxylin. (B1) Evaluation of collagen deposition per area (*p* = 0.04). (B2) There was no difference between the groups in the type I collagen (*p* = 0.73). (B3) There was a significant increase in type III collagen in the EG compared to the CG (*p* = 0.0338). The results were expressed as the mean ± SD.

At the site of the burn, there was stronger collagen deposition in the EG animals, which were treated with ADSC, than in the untreated CG animals (1.281 × 10^6^ ± 0.584 × 10^6^ vs. 0.680 × 10^6^ ± 0.661 × 10^6^, *p* = 0.04). For type I collagen, there was no significant difference between the EG and CG animals (14,242 ± 10,216 vs. 12,917 ± 6,998, *p* = 0.73); however, for type III collagen, the EG animals showed a higher deposition than the CG animals (4,886 ± 3,657 vs. 2,013 ± 1,401, *p* = 0.0338). The intensity of collagen deposition in the scar tissue was compared on the 14th day of burn evolution via the Masson trichrome histochemical method (Figure 6).

## 4. Discussion

Experimental studies indicated that the use of exogenous ADSCs led to distinct alterations in the cascade of events in each cicatricial phase [4]. In this work, to stimulate specific actions that affected distinct phases of the cicatricial process, the first injection of the ADSC was performed at the beginning of the inflammatory phase, and the second transplant occurred during the transition from the inflammatory phase to the proliferative phase, since the benefits of the transplant depend on the moment, the amount and site of cell infusion. Recommendations for the number of transplanted cells range between 1 × 10^6^ and 3 × 10^6^ cells [12]. In contrast, Bliley et al. transplanted 6.8 × 10^6^ ADSCs after twenty-four hours of the lesion and confirmed the survival of these cells until the twenty-first day of evolution [13]. In this work, 3.2 × 10^6^ ADSCs were transplanted around the burn immediately after the burns, and 3.2 × 10^6^ ADSCs were injected on the fourth day of evolution, with the intention of promoting ADSC effects in different cicatricial phases; the transplants led to effective results, with a marked reduction in the size of the scar area in the evaluated period.

The use of autologous MSCs in burns resulted in more accelerated cicatrization than the use of allogeneic MSCs. Burns are acute injuries in which the temporal evolution is relevant; in this way, allogeneic MSCs could be the only immediately available option [5]. In this research, allogeneic ADSCs were used for injection, considering that it is possible to obtain an abundance of adipose tissue with differentiation capability, genetic stability, and low senescence over a short period of time, demonstrating the possibility of a relatively fast and efficient growth [14].

Motamed et al. created burns on the back of rats by contact with a 20 × 55 mm metal bar heated in boiling water for thirty seconds and measured the surface of the burns on the seventh and the fourteenth days. Compared to the initial lesion size, the lesion size decreased by 42.2% and 84.9% in the ADSC transplant group, while in the control group, the reduction was 16.5% and 69.7% [15]. We also showed a statistically significant reduction in the burn size on the fourteenth day relative to the initial area in both groups, with the size in the CG decreasing 32.26% and that in the EG decreasing 40.44%. The reduction in the area of scar tissue was adequate in both groups, but compared to the CG, the EG presented a statistically significant reduction.

In the evaluation of Condé-Green et al., ADSCs improved the results by promoting evolutionary changes in the remodeling phase with increased fibroplasia [16]. Corroborating this research, at the beginning of the remodeling phase, a significant difference in fibrosis was found, with an intense reduction in the group that received ADSCs in the inflammatory and proliferative phases of healing.

In a healthy skin, type I collagen represents 80% of collagens, whereas collagen type III corresponds to only 10%. In the healing process, remodeling depends on the deposition of collagen type III, which increases at the beginning of repair until between the second and third weeks. After the lesion closes, collagen renewal begins with the degradation of type III collagen and the synthesis of type I collagen. In the healing evaluation period in this study, the significant increase in type III collagen in the EG was possibly due to ADSCs disrupting the regulation of growth factors, and this change, along with the greater number of collagen type III molecules, facilitated the formation of the matrix, contributing to the early reduction in scarring [17].

MSC injection at the edges of burns could modulate inflammation and increase VEGF even without flowing through the bloodstream [18]. The ADSCs incorporate into the capillary walls, enhance vascular network formation and recruit endothelial cells, and increase the amount of granulation tissue [19]. According to Tan et al., the interest in using MSCs for burn healing arises from their ability to induce neovascularization [20]; however, contrary to the findings of these authors, in this study, no difference was found between the groups in relation to the number of blood vessels at the cicatricial time investigated

As the cicatricial process becomes more finalized over time, the need for lymphatic vessels to remain decreases. It was observed that CO_2_ laser-induced burns in the inflammatory phase exhibited luminal diameter dilation and a temporary and heterogeneous increase in the density of lymphatic vessels with a temporary heterogeneous distribution and that there was a reversal of these changes [21], a phenomenon similar to that observed in this research.

In situations of edema, the lymphatic system benefits from lymphangiogenesis; therefore, to stimulate the inflammatory phase until the edema is resolved, lymphangiogenesis would be an efficient therapeutic modality. Insufficient lymphatic flow is associated with interstitial fluid accumulation and fails to transport immune cells from the periphery to the lymph nodes for antigen recognition [18]. In situations of extracellular matrix edema, the anchor ligaments open the lymphatic vessels, generating a unidirectional flow into the vessel, and despite their lack of smooth muscle cells, the vessels drain the lymph, cells, and debris [22]. The approaches for stimulating lymphangiogenesis should be controlled by the balance between prolymphangiogenic and antilymphangiogenic cytokines. Lymphatic vessels are important during healing because of their physiological regulation of the tissue pressure and their function as vectors and mediators of the immune response [19], which corroborates the results of this research showing an early return to local homeostasis with a reduction in the size of the scar tissue, possibly by an efficient and selective drainage of the edema generated by the burn.

It was found in this work that ADSCs can reduce the size and probably the healing time of burns in Wistar rats. In the EG, there was a predominance of the characteristics of the remodeling phase with increased collagen deposition, which indicates functional and aesthetic scarring with little fibrosis. A reduction in lymphatic vessels was observed on the 14th day of burn evolution, indicating a return to tissue homeostasis, possibly through the efficient modulation of the interstitial fluid drainage and the ability to resolve local inflammation, although the quantitative measurement of the cells showed no difference between the groups. One hypothesis to explain this lack of difference is based on the fact that the functional quality of each inflammatory cell was not considered at that scarring moment.

The abovementioned hypotheses require further research to search for concrete and significant evidence; however, the literature points to ADSCs as a consistent alternative for optimizing the cicatricial process, and this possibility has already led to excitement in the media, with healthy donors potentially undergoing a liposuction procedure to obtain ADSCs, similar to the process of donating blood to obtain its derivatives [23]. MSC injection is very promising in science research because of its demonstrated benefits in burn scarring in terms of quality, time, and modulation of inflammation [24]. However, to utilize these characteristics, experimental and clinical research should focus on safety, quality, transplantation predictability, and control of cellular reproducibility, as well as evaluate the safety and efficacy of ADSCs in clinical trials of cutaneous applications, neural regeneration, and chronic vascular ulcers. New research should broaden the understanding of the ADSC mechanisms of action and long-term fate and provide evidence of its efficacy in randomized trials [25].

## 5. Conclusions

The analysis of the results obtained in this study allows us to conclude that the ADSC injection influenced the cicatricial process in total skin thickness burns, as indicated by the measurements on the fourteenth day after the burn. Although the groups were similar in terms of inflammatory infiltrate and number of blood vessels, it was verified that the group that received the ADSC injection showed more collagen type III deposition and reductions in the cicatricial area and number of lymphatic vessels.

## Data Availability

The main data of immunophenotyping (flow cytometry) and histological sections (microscopy) used to support the findings of this study are included within the article.
